# Supplementary figures and images for: ACE2-lentiviral transduction enables mouse SARS-CoV-2 infection and mapping of receptor interactions
Source: PLoS Pathog. 2021 Jul 2;17(7):e1009723. doi: 10.1371/journal.ppat.1009723 (PMC8282004; doi:10.1371/journal.ppat.1009723)

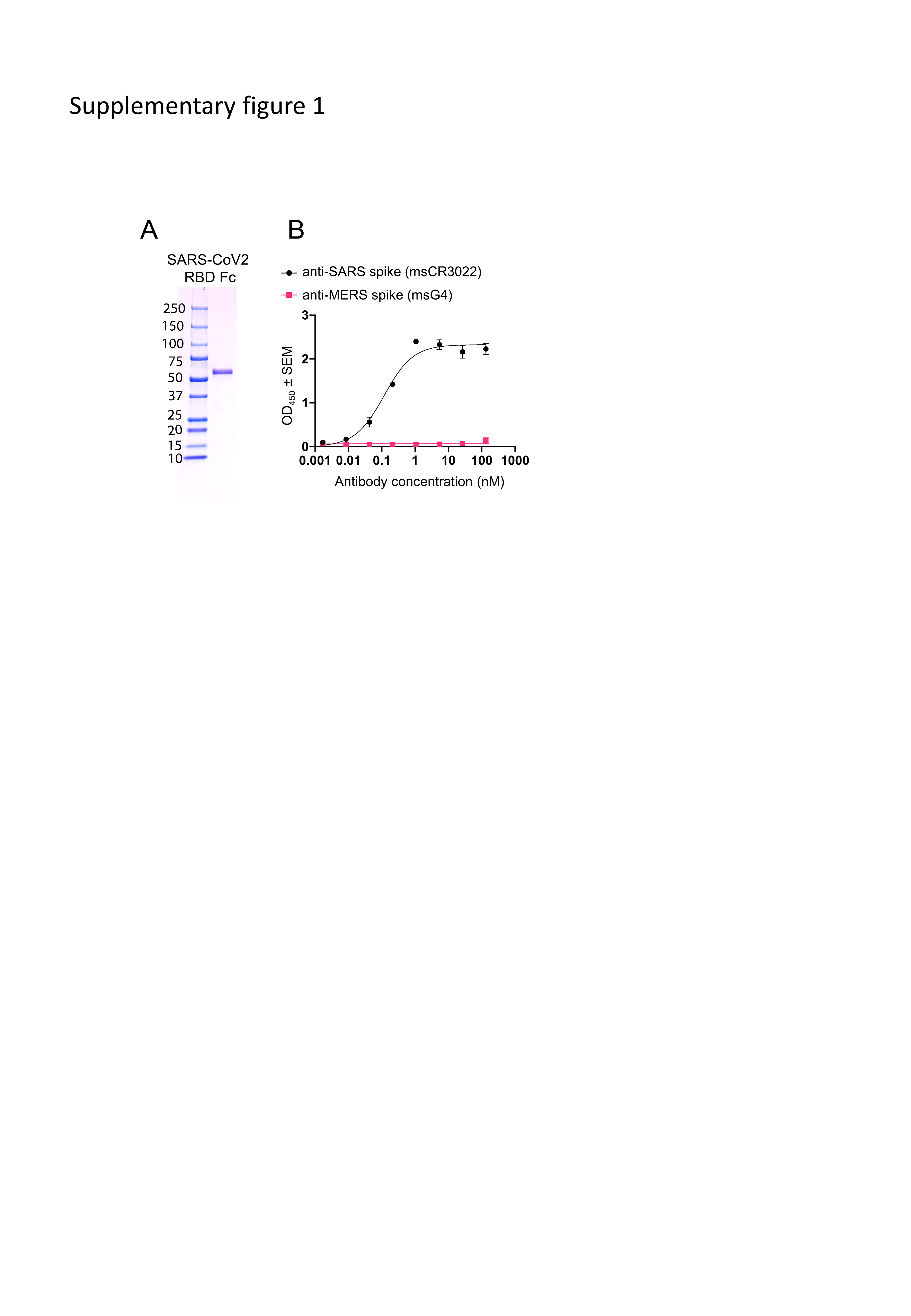

Supplement: S1 Fig — A) Recombinant SARS-CoV-2 spike RBD-Fc purity was analyzed on reducing SDS-PAGE followed by Coomassie staining and B) folding validated using recombinant mouse anti-SARS spike antibody (msCR3022) or mouse anti-MERS spike antibody (msG4) in ELISA. (TIF) [file ppat.1009723.s001.tif]

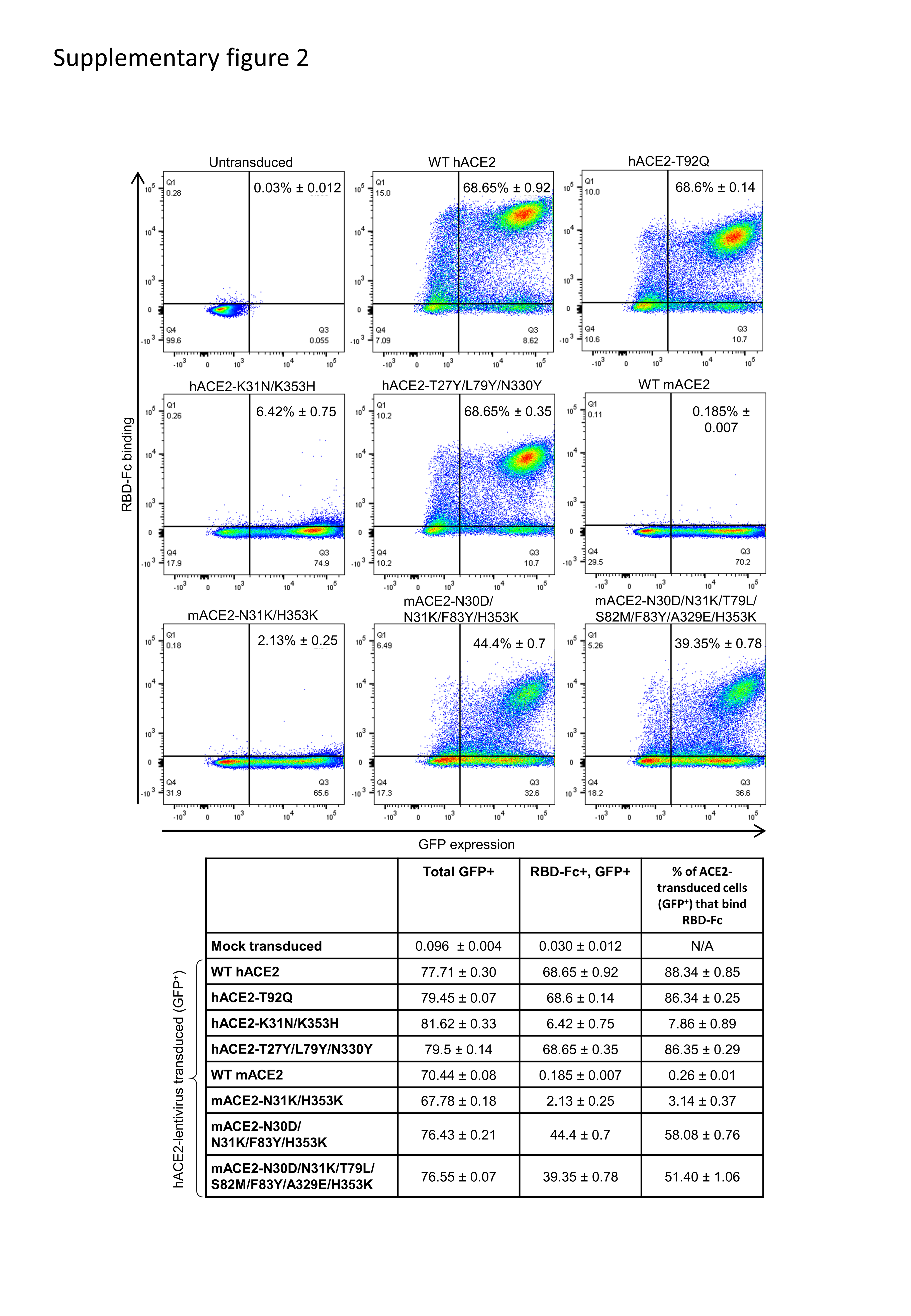

Supplement: S2 Fig — Flow cytometry gating of RBD-Fc binding (AlexaFlour 594, y-axis) to ACE2-transduced (GFP+, x-axis) cell lines. Values are given in the table for the total percentage of cells expressing GFP, and GFP+ cells with RBD-Fc bound. n = 2–3 per cell line, with an independent repeat showing similar results. (TIF) [file ppat.1009723.s002.tif]

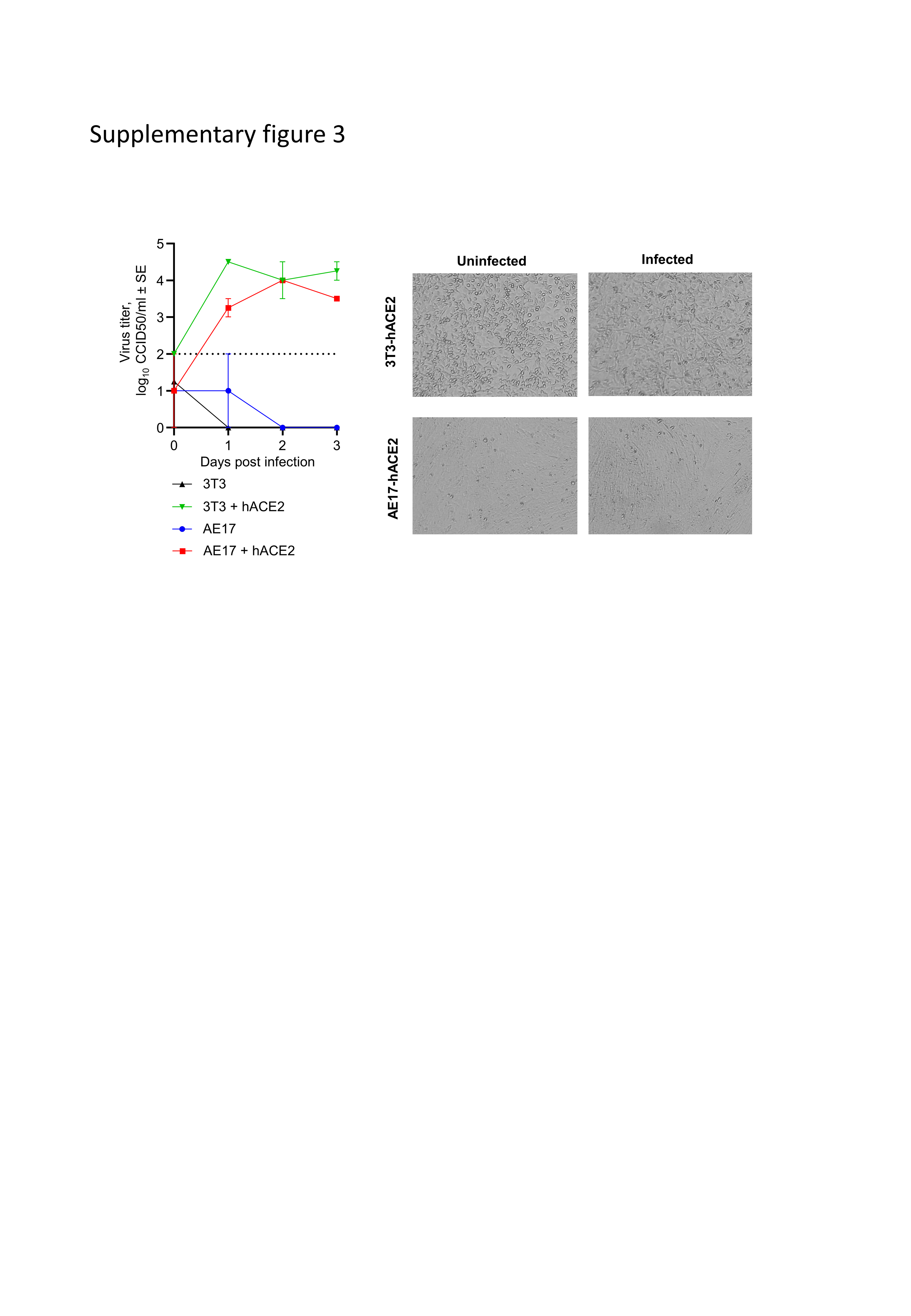

Supplement: S3 Fig — Growth kinetics of SARS-CoV-2 over a three day time course in mock transduced or hACE2-lentivirus transduced 3T3 or AE17 cells infected at MOI = 0.1. Data is the mean of triplicate wells and error bars represent SEM. Images of cells were taken using an inverted light microscope at day 3 post-infection and are representative of triplicate wells. (TIF) [file ppat.1009723.s003.tif]

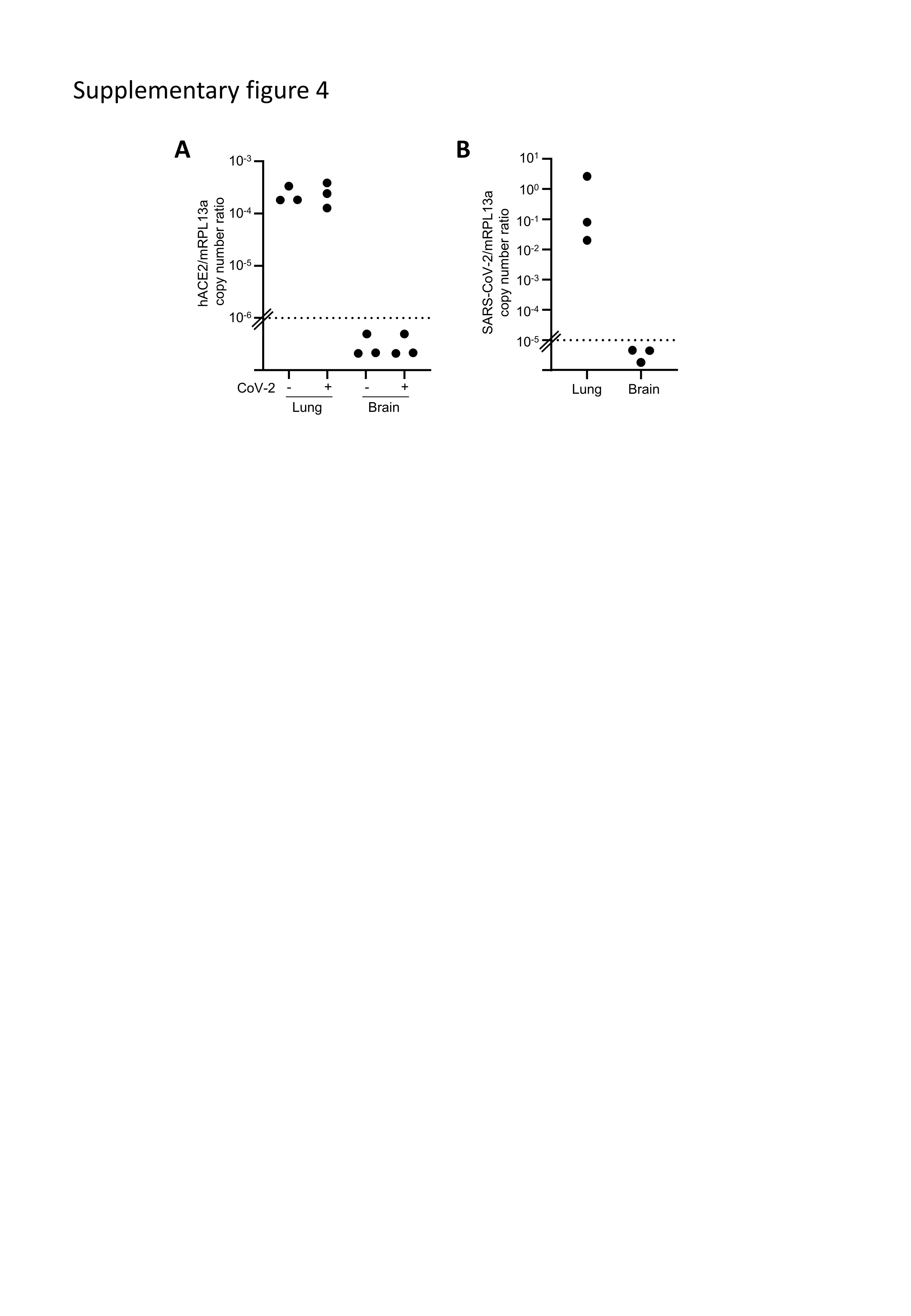

Supplement: S4 Fig — A) RT-qPCR of mouse lung and brain RNA using primers for hACE2 gene normalized to mRPL13a levels. Data is individual mice (n = 3 per group) and is expressed as RNA copy number ratio calculated using a standard curve. Horizontal line indicates cut-off for reliable detection. B) RT-qPCR of mouse lung and brain RNA on day 4 post-infection using primers for SARS-CoV-2 E gene normalized to mRPL13a levels. Data is for individual mice and is expressed as RNA copy number ratio calculated using a standard curve. Horizontal line indicates cut-off for reliable detection. (TIF) [file ppat.1009723.s004.tif]

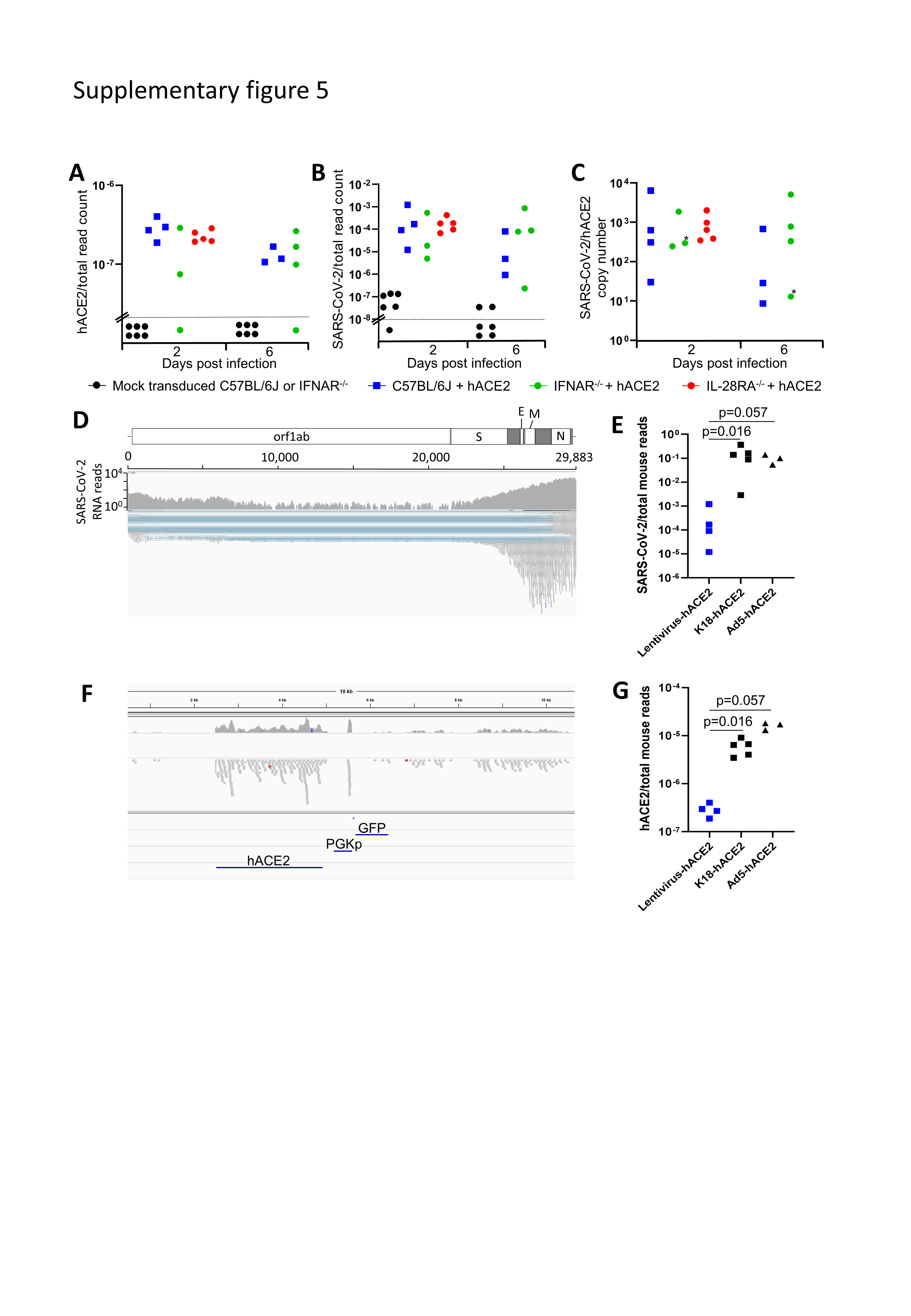

Supplement: S5 Fig — RNA-Seq was performed on mouse lung from the same mice as in ‘Fig 2‘. A) hACE2 read counts normalized to total read count. Data points below the horizontal dotted line had read counts of zero. B) SARS-CoV-2 read counts normalised to total read count. Data points below the horizontal dotted line had read counts of zero. C) SARS-CoV-2 read count normalised to hACE2 read count. Circle with asterisk had hACE2 read count of 0, so the value was set to the SARS-CoV-2 read count (not normalised). D) SARS-CoV-2 reads aligned to reference genome viewed in Integrative Genome Viewer (IGV). Mice with hACE2 displayed reads mapped across the entire genome, with higher counts for structural gene sub-genomic RNA as also evident in hACE2-adenoviral vector transduced cell lines [64]. E) SARS-CoV-2 read counts normalised to total mouse reads from RNA-seq data for lentivirus-hACE2 transduced mice at day 2, K18-hACE2 transgenic mice at day 4, and downloaded data for Ad5-hACE2 transduced mice at day 2 [53]. Statistics by Kolmogorov Smirnov test. F) hACE2-lentivirus reads aligned to the lentivirus reference genome viewed in IGV. RNA from hACE2-lentivirus transduced mouse lung had reads mapping across the entire hACE2 gene, but did not have reads mapping across GFP. G) hACE2 read counts normalised to total mouse reads from RNA-seq data for lentivirus-hACE2 transduced mice at day 2, K18-hACE2 transgenic mice at day 4, and downloaded data for Ad5-hACE2 transduced mice at day 2 [53]. Statistics by Kolmogorov Smirnov test. (TIF) [file ppat.1009723.s005.tif]

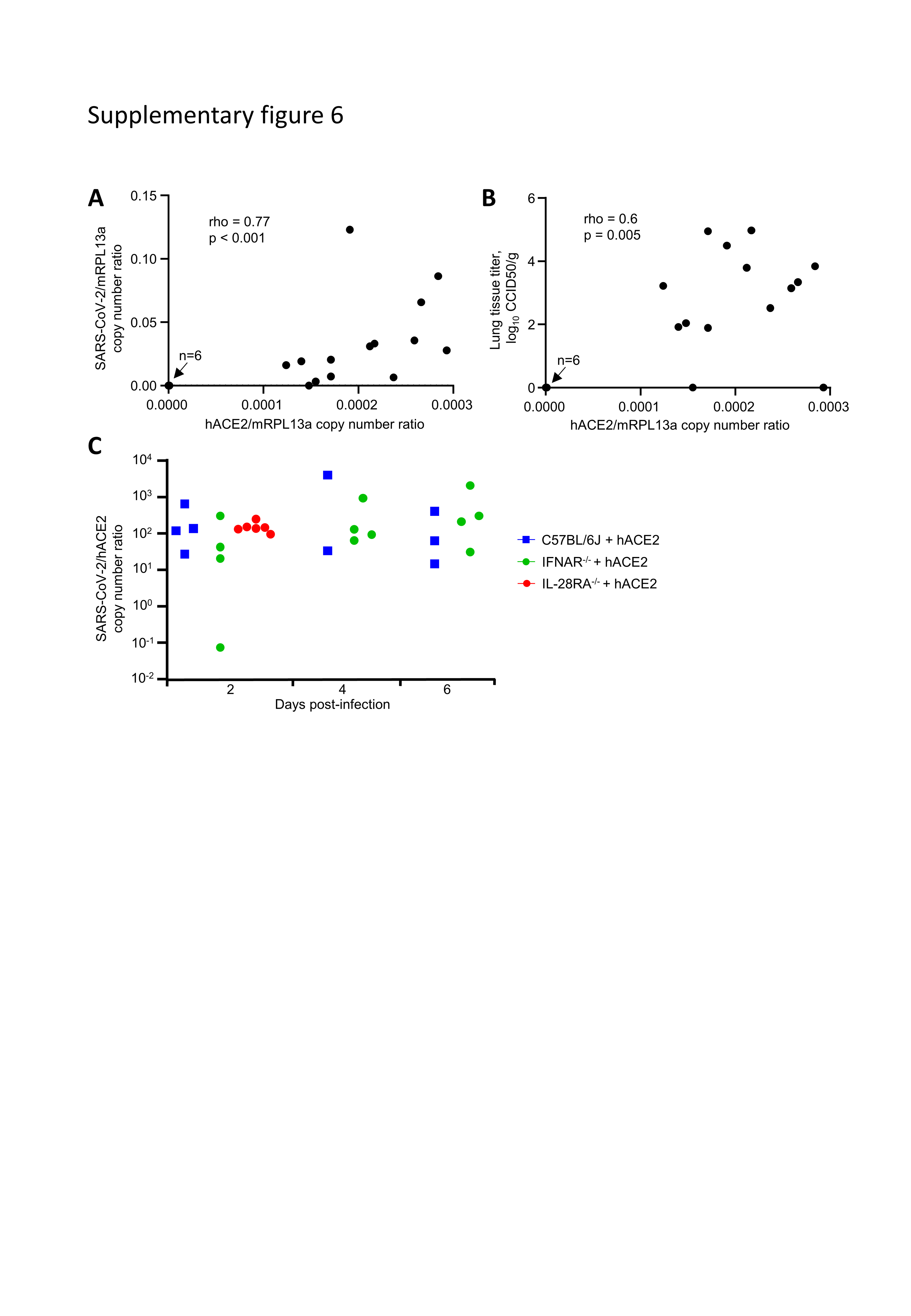

Supplement: S6 Fig — A) Correlation of SARS-CoV-2/mRPL13a copy number ratio from Fig 2E with hACE2/mRPL13a copy number ratio from Fig 2C). Correlation is significant by Spearman correlation. B) Correlation of SARS-CoV-2 lung tissue titer from Fig 2D with hACE2/mRPL13a copy number ratio from Fig 2C). Correlation is significant by Spearman correlation. C) RT-qPCR of mouse lung RNA using primers for SARS-CoV-2 normalized to hACE2 introduced by lentivirus transduction. Data is individual mice from Fig 2E normalised to Fig 2C, and is expressed as RNA copy number calculated against a standard curve for each gene. (TIF) [file ppat.1009723.s006.tif]

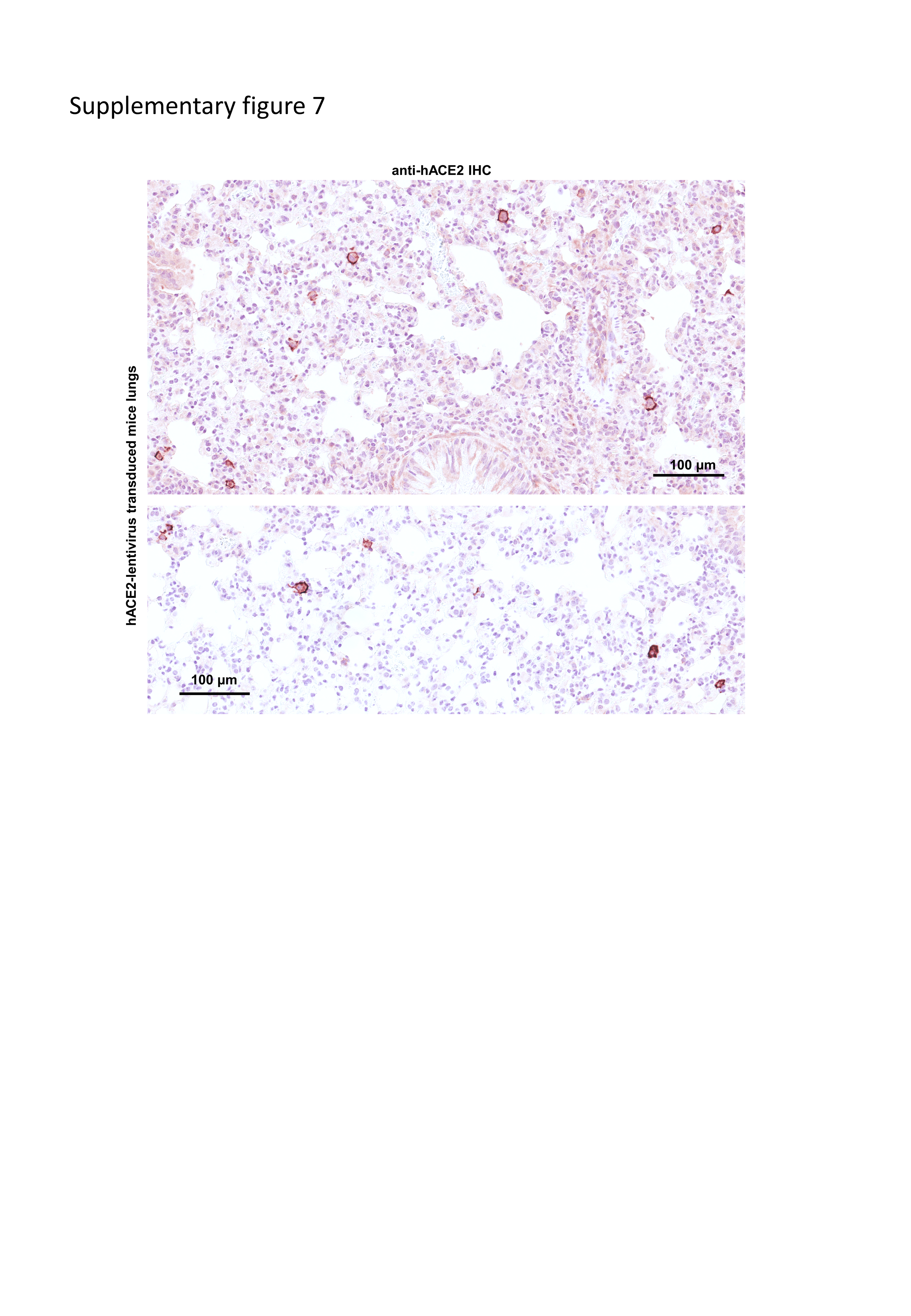

Supplement: S7 Fig — Examples of anti-hACE2 IHC (brown staining) in mouse lung alveolar epithelial cells, additional to Fig 3G. Staining was evident in mouse lungs transduced with hACE2-lentivirus, and no staining was evident in mock transduced mouse lung. (TIF) [file ppat.1009723.s007.tif]

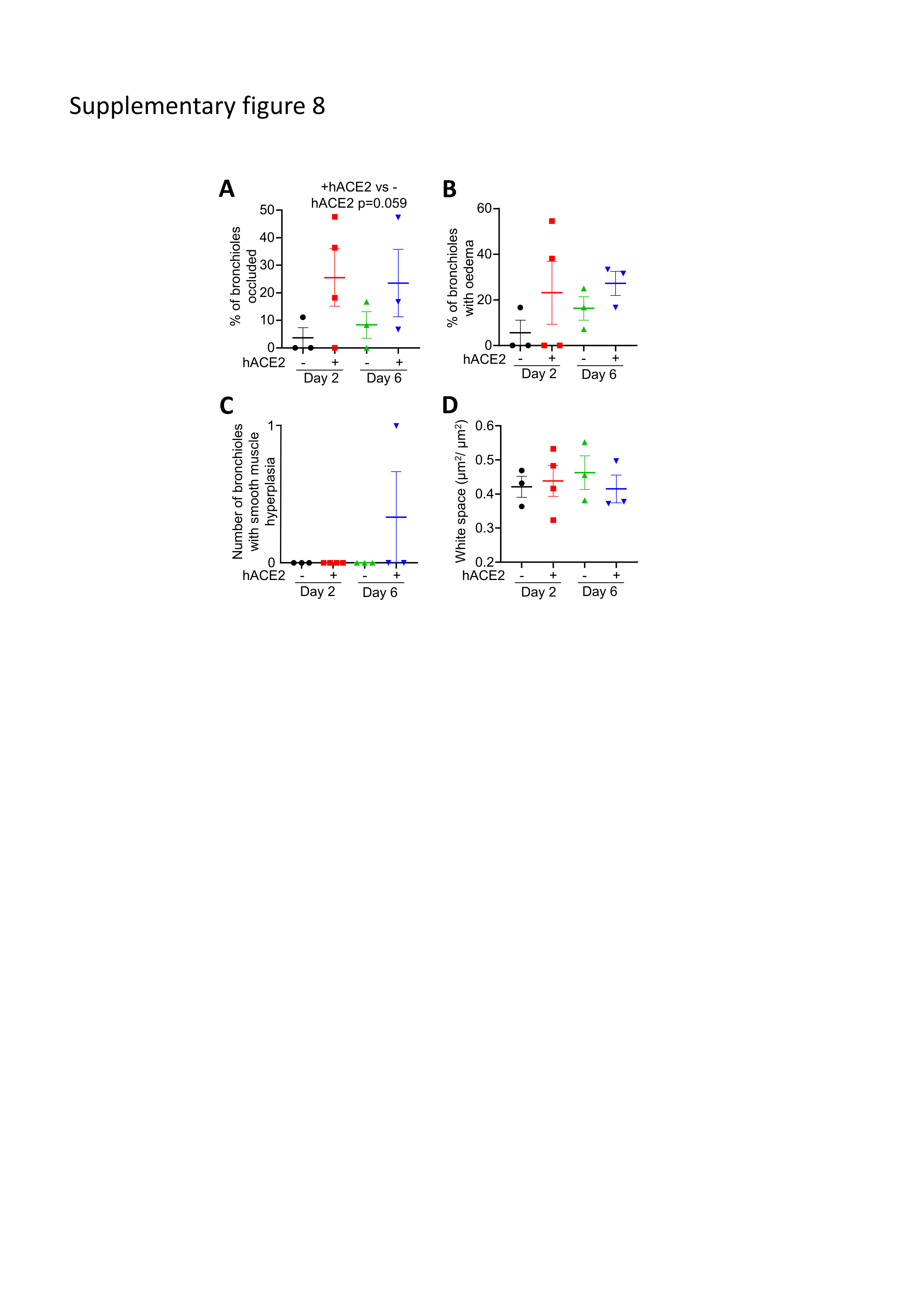

Supplement: S8 Fig — A) Occluded bronchioles were determined based on substantially reduced space within the bronchiole, either due to oedema or complete collapse, indicating occlusion. The percentage of bronchioles occluded as a proportion of total bronchioles for each mice is shown. When all hACE2-transduced mice are compared to mock transduced mice, both of which received an intra-lung inoculum of SARS-CoV-2, the percentage of bronchioles occluded approaches significance by Kruskal-Wallis test. B) Bronchioles with oedema were counted when evidence of fluid was present within the bronchiole. The percentage of bronchioles with oedema as a proportion of total bronchioles for each mice is shown. C) Smooth muscle hyperplasia was counted based on a muscle expansion greater than naïve mouse lung. Only 1 bronchiole (day 6 + hACE2-lentivirus) of all lungs examined had clear evidence of smooth muscle hyperplasia. D) Automatic quantitation of white space was undertaken using QuPath v0.2. n = 3–4 per group for all analyses. (TIF) [file ppat.1009723.s008.tif]

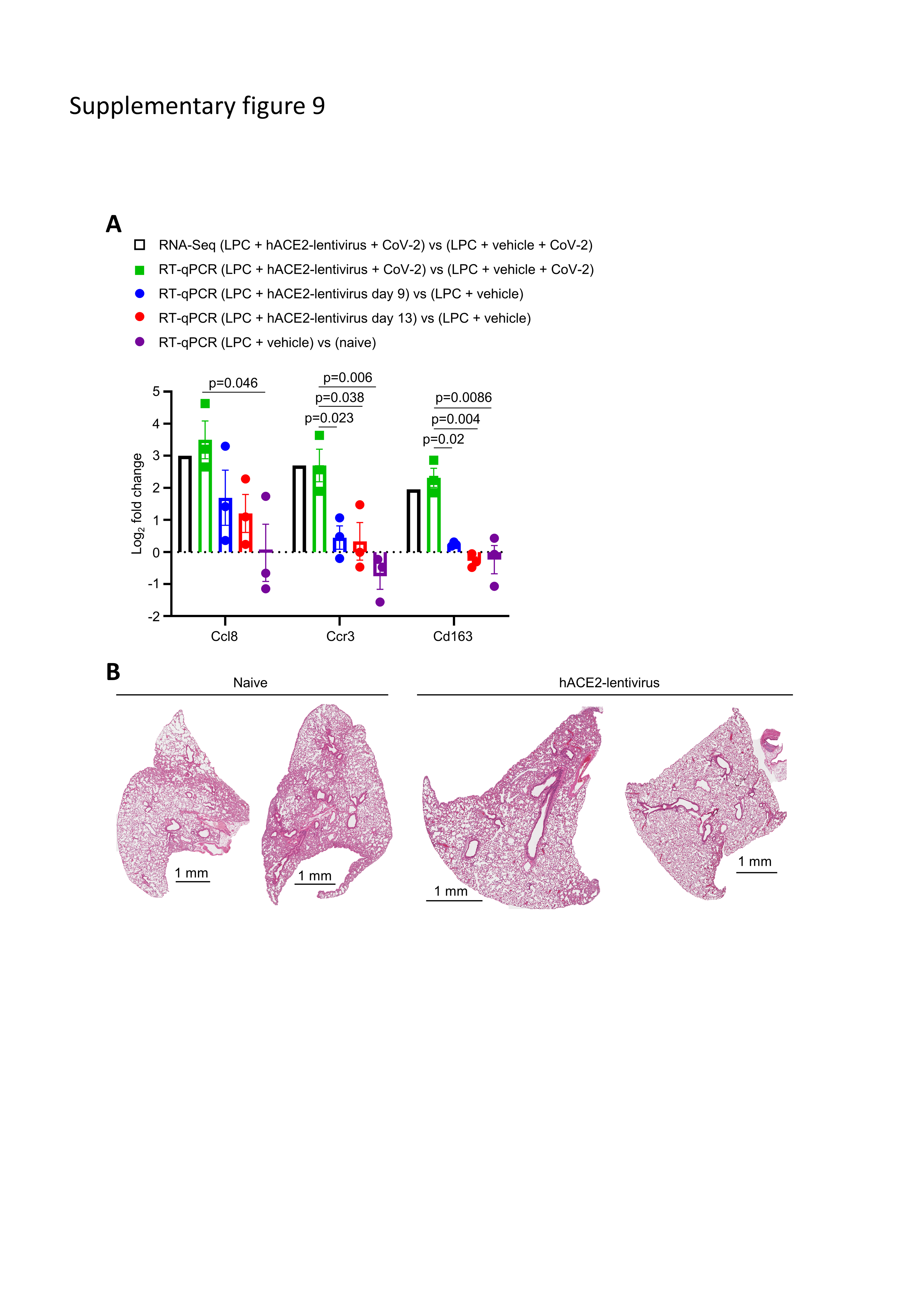

Supplement: S9 Fig — A) Log2 fold change for Ccl8, Ccr3 and Cd163 determined by RNA-Seq (S1C Dataset) is represented by the black bar. Log2 fold change for Ccl8, Ccr3 and Cd163 was calculated from RT-qPCR data using the 2-ΔΔCt method for the indicated comparisons. RT-qPCR for Ccl8, Ccr3 and Cd163 using the same RNA samples used in RNA-Seq validates both assays. Statistics are by t-test and shows significantly higher induction of Ccl8 in SARS-CoV-2 infected mice (green) compared to LPC plus vehicle only (purple), and significantly higher induction of Ccr3 and Cd163 compared to hACE2-lentivirus transduction only (blue and red). B) H&E stained lung sections from naïve mice (left) and mice that received intrapulmonary LPC + hACE2-lentivirus harvested at 9 days post-inoculation (right). (TIF) [file ppat.1009723.s009.tif]

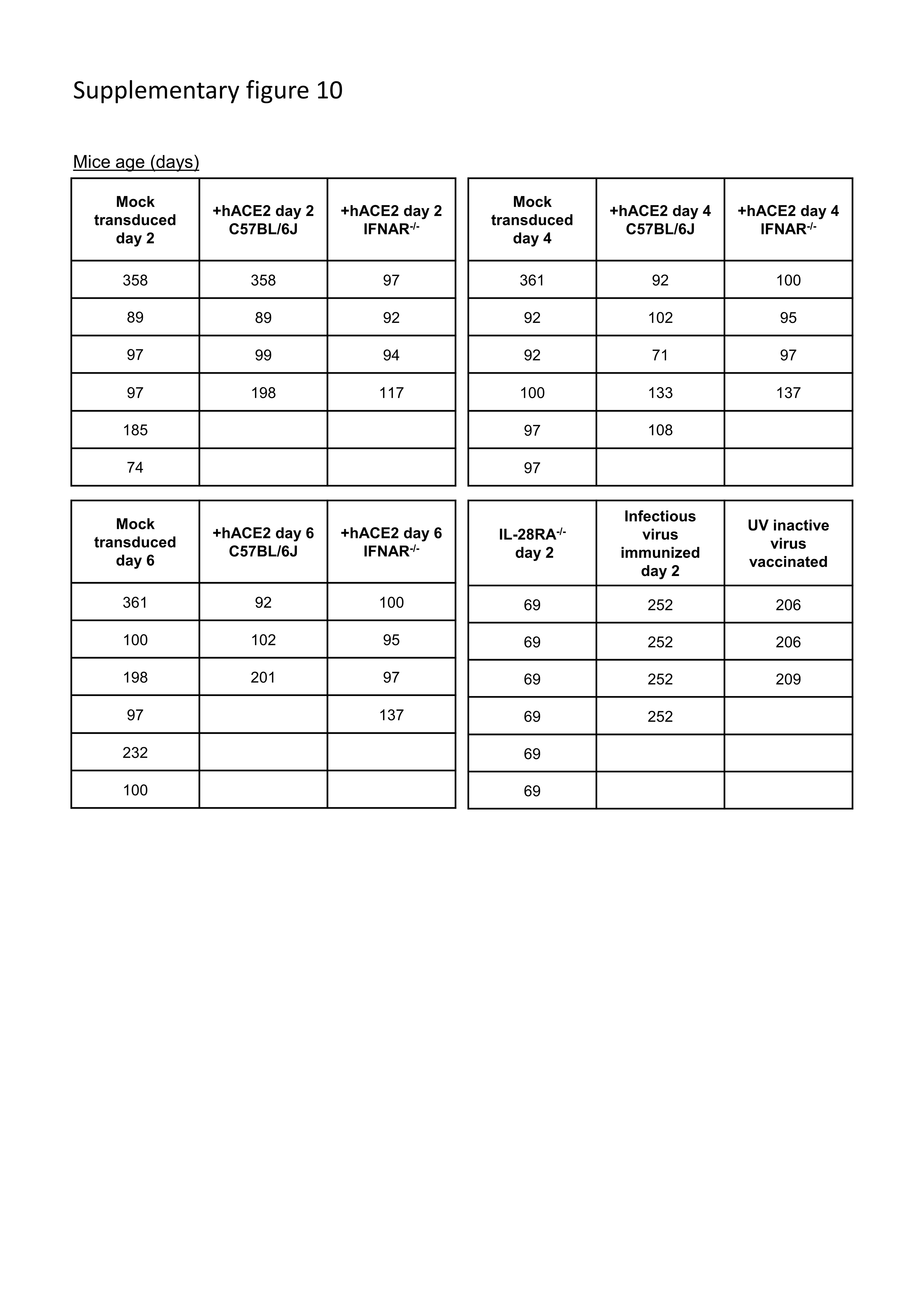

Supplement: S10 Fig — Individual mouse ages are shown in days at the time of SARS-CoV-2 inoculation. (TIF) [file ppat.1009723.s010.tif]
