# Supplementary material for: ACE2-lentiviral transduction enables mouse SARS-CoV-2 infection and mapping of receptor interactions
Source: PLoS Pathog. 2021 Jul 2;17(7):e1009723. doi: 10.1371/journal.ppat.1009723 (PMC8282004; doi:10.1371/journal.ppat.1009723)
Supplement: Attachment — Submitted filename: Lenti_rebuttal_v3.docx [file ppat.1009723.s016.docx]

Reviewer's Responses to Questions. All the reviewers comments have been recapitulated below to facilitate re-review.

Author responses in yellow. **Bold text refers to inclusion of new data.**

**Part I - Summary**
Please use this section to discuss strengths/weaknesses of study, novelty/significance, general execution and scholarship.

Reviewer #1: This study describes a lentiviral transduction system for the expression of hACE2 in cell lines and mice. The utility of the model is clearly displayed for evaluating ACE2 residues important for in vitro replication, and the structure models are presented clearly. The study is well-written and the results are clearly described. However, there is a heavy emphasis on gene expression analysis that is of questionable relevance to human disease given the limited replication observed in the model.

*We thank the reviewer for their comments. We agree there was a heavy emphasis on gene expression analyses, although these analyses do cover several different important comparisons (-hACE2 vs +hACE2, day 6 vs day 2, C57BL/6J vs IFNAR^-/-^, C57BL/6J vs IL-28RA^-/-^ and vaccinated vs unvaccinated). We compare inflammatory responses in these mice with that of human disease, and show significant similarities in gene regulation (Figure 4F). However we have de-emphasized gene expression analysis overall by* ***including new H&E histology (Figure 3, Figure 6F-H, Figure 7G and Supplementary Figure 8), IHC for hACE2 and SARS-CoV-2 (Figure 2G and H, and Supplementary Figure 7), and ACE2-RBD binding assay data (Figure 1E, Supplementary Figure 1 and 2)****. Please see further responses to specific points below.*

Reviewer #2: This manuscript by Rawle et al., describes evaluation of a lentiviral vector encoding human ACE2 receptor as a means to sensitize diverse mouse strains to infection with human SARS-CoV-2 viruses. The approach is a useful contribution to the field which will complement existing studies which have evaluated transgenic hACE2 mouse models, and other viral vector mediated models in which hACE2 can be transiently delivered to any mouse strain or genetic knockout using adenoviral or adeno-associated viruses. Each model has its pros and cons. Therefore, detailed descriptions of the utility of each model will be informative to investigators in the field. This manuscript has shown that lentiviral delivery of hACE2 to mouse lung is an effective means of sensitizing C57Bl6, IFNAR-/- and IL-28RA-/- mice to SARS-CoV-2 infection. The authors also shed light on the residues within the SARS-CoV-2 receptor binding domain (RBD), as well as human and murine ACE2 which contribute to the specificity of binding and ability to use ACE2 as an entry receptor. In addition, the authors provide a comprehensive evaluation of genes which are differentially expressed following challenge in mice, and compare these data sets with existing literature for both the K18-hACE2 transgenic mouse model, and human data sets. This is a nice validation of the model.

In general, the manuscript is easy to follow, however there are several sections which require clarification and additional detail. Some jargon and acronyms for gene expression data could be clarified to make the figures more accessible, and to make the context apparent to non-expert readers. There are some discrepancies in figures which need to be addressed and additional experiments are needed. The model would benefit from the addition of histology to confirm the spatial distribution of hACE2 expression following lentivirus transduction, as well as H&E to visualize lung pathology post-challenge. These are important parameters for establishing the model when compared with existing studies.

*We thank the reviewer for their comprehensive comments.* ***H&E (Figure 3, Figure 6F-H, Figure 7G and Supplementary Figure 8) and IHC (Figure 2G and H, and Supplementary Figure 7) has now been included.*** *Please find specific responses to all of these comments below.*

Reviewer #3: Andreas Suhrbier and colleagues submitted a well written manuscript that details the development of a lentivirus expression system for ACE2. Lentivirus transduction of mouse airway with human ACE2 conferred susceptibility to infection with SARS-CoV-2. However, infection of the lentivirus transduced mice resulted in minimal body weight loss. This observation suggests that although gene delivery of hACE2 promoted virus replication resulting in detectable infectious virus titers, the level of virus replication was still restricted. Immunohistochemistry (IHC) to detect transduced GFP or hACE2 and viral antigen would provide supportive data for the limited body weight changes. These IHC data would also help with interpretation of the RNAseq datasets in terms of location of viral replication and infected cells.

*We thank the reviewer for their comments.* ***IHC for hACE2 and SARS-CoV-2 has now been included (Figure 2G and H, and Supplementary Figure 7).*** *Please see further responses to specific points below.*

The authors identify four amino acid residues within mouse ACE2 that are necessary for infection by SARS-CoV-2; however, the authors did not experimentally examine if the changes altered binding affinity.

***New data from binding assays have now been included (Figure 1E, Supplementary Figure 1 and 2).*** *Remarkably, mutations that did not significantly affect virus replication had significant effect on RBD-ACE2 binding. We have added a new results section: “RBD binding assays indicate low levels of receptor binding allow virus replication”* ***(lines 136-158).***

The investigative team utilized their model system to examine the role of type I and type III interferon pathways in respiratory disease resulting from SARS-CoV-2 infection. Although the lack of either interferon pathway did not alter the kinetics or magnitude of virus replication, the authors did note from their RNA-seq data differences in differentially expressed genes, which suggests that these interferon pathways target distinct subsets of genes.**Part II – Major Issues: Key Experiments Required for Acceptance**
Please use this section to detail the key new experiments or modifications of existing experiments that should be absolutely required to validate study conclusions.

Generally, there should be no more than 3 such required experiments or major modifications for a "Major Revision" recommendation. If more than 3 experiments are necessary to validate the study conclusions, then you are encouraged to recommend "Reject".

Reviewer #1: Major points
1) What cells does the lentivirus transduce in vivo? If this is known in the literature it should be referenced. If not staining for hACE2 expression in the lung should be included.

***We have now provided hACE2 immunohistochemistry in lentivirus transduced mice lungs (Figure 2G and Supplementary Figure 7).*** *This showed that intrapulmonary hACE2-lentivirus transduced alveolar epithelial cells, which is consistent with the literature and this is now referenced in the text* ***(line 78-81)****. We have added a new results section: “hACE2 and SARS-CoV-2 localize to alveolar epithelial cells in hACE2-lentivirus transduced mouse lungs”.*

Also does the lentivirus induce any measurable inflammation and at what time points?

***We have now included RT-qPCR for genes identified as upregulated in SARS-CoV-2 infected lungs by RNA-Seq (Supplementary Figure 9).*** *The new data shows that LPC + hACE2-lentivirus transduction did not significantly induce upregulation of these genes, indicating that the inflammatory signature after SARS-CoV-2 infection is specific for virus replication.* ***This is also supported by new H&E histology analyses (Supplementary Figure 9). See lines 291-297.***

2) Experiments in Fig 1B need to be repeated at least one time and preferably 2 to determine if they are reproducible.

***We have repeated growth kinetics*** ***in all hACE2 and mACE2 mutant cell lines an additional 1-2 times.*** *There is now 2-4 independent experiments with 2-3 replicates in each. The data was reproduced in the additional repeats and the conclusions remain unchanged.*

Similarly, some of the groups in figure 2 only have 2 animals, this is problematic given the large spread in the results.

*Only 1 group had 2 animals, C57BL/6J + hACE2 at 4 dpi. C57BL/6J at 6 dpi had 3 mice, while all other groups had at least 4 mice.* ***We have now repeated the experiment using an additional 3 mice harvested at day 4 post infection and this additional data is included in Figure 2.***

RNA copy number does not correlate well at day 2 when replication is detected, this is surprising given how consistent hACE2 expression levels are, so some explanation should be provided for these results.

*We have now included a correlation of hACE2 with SARS-CoV-2: “As might be expected, viral RNA levels (measured by RT-qPCR or titer) showed a highly significant correlation with hACE2 RNA expression levels* ***(Supplementary Figure 6A and B)****.” However, the higher variability in virus replication is now discussed in the limitations section of the discussion* ***(see lines 505-526)****.*

3) In my opinion the differential gene expression analysis doesn’t warrant 3 full figures. While there is some useful information here, because viral replication in this model is limited to the few cells that take up the lentivirus, the relevance of this data to human disease is not clear. Also given that replication is limited to D2 post-infection, its again unclear the relevance of the D6 tissue repair signatures. Likewise, loss of IFN-related gene upregulation in IFNAR-deficient mice is not surprising.

*We have moved some RNA-Seq panels from the main figures 6 (IFN) and 7 (vaccine) to supplementary tables* ***and replaced with histological analyses (Figure 3, Figure 6F-H and Figure 7G).*** *This new analyses implicates day 6 in having specific histological lesions, and shows that type I IFN was needed for significant leukocyte infiltration. We thus think it is important for these gene expression analyses figures to remain as main figures, however these are now de-emphasized by inclusion of more non-RNA-Seq data as described above.*

These experiments would have been better controlled by transducing the control group with empty or irrelevant lentivirus, to control for any inflammation induced by the lentivirus or lysophosphatidylcholine.

*Lysophosphatidylcholine was administered to all mice, and this has now been made explicit in the text and figure 2. Empty lentivirus poses different problems, as it is usually has much higher transduction efficiency and expression levels, potentially over-correcting any lentivirus responses. Arguably mACE2 lentivirus may be the most appropriate control, however this was not available at the time of the animal experiments used for RNA-Seq.* ***We have now shown by RT-qPCR and histology that there is no obvious inflammation induced by hACE2-lentivirus alone (Supplementary Figure 9)****. Lentiviruses are known to be much less immunogenic than adenoviruses, and this is now referenced in the limitations sections of the discussion* ***(see*** ***lines*** ***505-526)****.*

Reviewer #2: Major comments
• Knowledge regarding the anatomical distribution of expression of hACE2 delivered by a lentiviral vector in the mouse lung would be informative to the field. As this study aims to contribute the utility of this SARS-CoV-2 sensitization model, information regarding the histological expression of hACE2 would be informative to investigators in deciding which model is most appropriate to their study.

***We have now provided hACE2 immunohistochemistry in lentivirus transduced mice lungs (Figure 2G and Supplementary Figure 7). Please refer to reviewer #1 major comment 1.***

In addition to adding histology (IHC staining for hACE2), the inclusion of H&E stained lung sections after SARS-CoV-2 challenge would demonstrate the “severity” of the model, and could verify the potential effect of inflammatory cell infiltration diluting RT-qPCR results (as stated on Page 13, Line 327-329 with regard to Fig.6C). The authors state on Page 10, Line 239 that severe disease in SARS-CoV-2 animal models is typically measured by weight loss, however this is not always the case as many models do not result in weight loss. Again, this supports the need to provide histological specimens to determine lung pathology/severity for this model.

***We have now added new H&E analyses (Figure 3, Figure 6F-H, Figure 7G and Supplementary Figure 8).*** ***We have added a new results section (see lines 266-279)****: “SARS-CoV-2 replication induces histopathological lesions”. This shows that there was more leukocyte infiltration and bronchiolar membrane sloughing on day 6 in mice that have hACE2.*

*We also show that IFNAR^-/-^ mice have less leukocyte infiltration* ***(Figure 6F-H, new results section “Type I IFN signaling drives leukocyte infiltration during SARS-CoV-2-induced lung inflammation”, see lines 422-430****),* *and vaccinated mice had higher infiltrates on day 2* *(****Figure 7G, see lines 454-455).***

• It is unclear if some of the same mouse groups have been used for data presented in Fig.2C and Fig.6C, untransduced and unvaccinated. The data points look very similar. Also, the same observation has been made for Fig.2E and Fig.6D for the same groups. If these were the same groups run for different arms of an experiment, this should be clearly stated in the text and in the figure legends.

*Yes, the data for untransduced and C57BL/6J in Fig. 2 and 6 are the same. The challenge in Fig. 7 was performed at the same time as the infection in Fig. 2 and thus the controls for this experiment is the same. We have now stated this explicitly in the text* ***(line 442-444)*** *and figure legend for Fig. 7.*

• More detail is required for the animal experiments. For example, group sizes are not defined and in Fig. 2, it appears that only an n=2 were analyzed for the C57Bl6 mice. Age and sex of animals should also be clearly outlined for routine strains and genetic knockouts.

*Group sizes have now been defined in the figure legends of Figure 2 and 7. All mice were female and aged between 8 weeks to 1 year old as stated in the methods section.* ***We have now provided Supplementary Figure 10 which shows each animal’s age.*** *Please see major comment #2 from reviewer #1 regarding the n=2 group of C57BL/6J mice at 4 dpi.*

Reviewer #3: The authors should perform immunohistochemistry to examine the pulmonary distribution of hACE2 gene delivery and assess the type and distribution of infected pulmonary cells.

***We have now provided hACE2 immunohistochemistry in lentivirus transduced mice lungs (Figure 2G and Supplementary Figure 7). Please refer to reviewer #1 major comment 1.*Part III – Minor Issues: Editorial and Data Presentation Modifications**
Please use this section for editorial suggestions as well as relatively minor modifications of existing data that would enhance clarity.

Reviewer #1: Minor points
1) Given that many of the mutations do not result in the predicted phenotypes in terms of increased/decreased replication, the results should be further discussed and placed in the context of the literature in terms of what is known about mouse adaptation and variants.

*A new discussion of the hACE2 mutant binding and virus replication data has been added to the discussion in* ***light of our new binding data (see lines 530-541)****. Reference to mouse adaptation and variants have been added to the results section* ***(see lines 179-182)****.*

2) You claim that T92Q doesn’t result in any change in SARS-CoV-2 replication, however there is no validation that the mutation results in a glycosylation change in these cells.

*We have now provided a reference that shows that N90 is glycosylated in HEK293 cells, which is the cell line we used in these experiments* ***(see lines 104-107)****. The T (or S) in an N-X-T motif is known to be required for N-linked glycosylation.*

3) The explanation of Fig 2F is confusing and I’m not sure the point you are trying to make. It may be more useful to present read numbers of hACE2 and SARS-CoV-2 in the different systems to see how they compare.

***Read numbers for total hACE2 and SARS-CoV-2 are now provided in Supplementary Figure 5E and G.*** *The point we are trying to make in Figure 2F is that while hACE2 levels are lower than transgenic mice and Ad5 transduction, replication efficiency (SARS-CoV-2/hACE2) is comparable. We have re-written this section and reference the new data* ***(see lines 230-235)****.*

4) Ln 351 – you claim a key advantage of lentiviruses is long term stable expression - while adenovirus does not integrate, it does induce long-term expression

*We have removed ‘long-term” from this sentence.*

Reviewer #2: Minor comments
• The authors should justify why an interval of 1 week between lenti-hACE2 administration and challenge was selected. Is this known to be the maximal peak for transgene expression following lung delivery?

*We have now justified this in the text* ***(see lines 206-209)****.*

• What is known about lentivirus off target effects following intranasal administration. When comparing to the K18-hACE2 model this would be useful to know. Are lentiviruses known to infect olfactory bulb or to gain access to the brain?

***We have now performed RT-qPCR for hACE2 and SARS-CoV-2 in brains of mice after intrapulmonary hACE2-lentivirus transduction and SARS-CoV-2 infection (Supplementary Figure 4).*** *This shows that mice brains are not transduced or infected in this model. The following has also been added to the discussion: “Brains of mice that received intrapulmonary hACE2-lentivirus inoculation had no detectable hACE2 or SARS-CoV-2 RNA, consistent with literature that suggest there is no transduction of olfactory epithelium following intrapulmonary lentivirus inoculation in mice (93), and microinjection directly into olfactory epithelium is required for transduction (94).”,* ***see lines 212-213and 464-468.***

• Page 6, Line 148-155. The authors should add some comment on the role of various proteases ie TMPRSS2, and their distribution in different cell lines, in affecting the ability for SARS-CoV-2 to replicate (PMID: 32142651).

*The suggested reference and others regarding the role of proteases in SARS-CoV-2 replication and how this may affect tropism in cell lines has now been added* ***(see lines 189-193)****.*

• It is not always explicitly clear if untransduced animals were also challenged with SARS-CoV-2. This should be more clearly stated throughout the manuscript (although it is noted that this is outlined on Page 8, Line 193).

*This is now clearly stated at the start of the results section describing the mice experiments* ***(see lines 204-205)*** *and in the relevant figures.*

• Figure.2F. No description in the methods is provided for the use of the Ad5-hACE2 transduction model as a comparator.

*We downloaded RNA-Seq data for a published Ad5-hACE2 publication and this is described in the methods* ***(see lines 1001-1008)****. We have now made this explicit when describing these results* ***(see lines 230-235)****.*

• Figure.3. A more detailed explanation of what is being analysed in 3D should be described in the figure legend. In addition, please indicate in Page 8, Line 205-206 if this was performed on SARS-CoV-2 infected lung.

*We have now provided more detail about this analyses in the results text* ***(lines 302-303)****, figure legend and we have* ***now provided these DEG lists in full our supplementary table 1G*** ***(lines 649-654)****.*

• A section describing the limitations of this model in comparison to others should be discussed.

*A limitations section is now provided in the discussion (****see lines 505-526)****.*

Page 3, Lines 75-78. Please provide specific information regarding the duration of expression, and the specific cell types transduced based on the existing literature. Putting this into context by comparing and contrasting against existing models for SARS-CoV-2 (ie. Adeno or AAV) would be useful to the field. It would also be informative to comment on if anti-vector immunity affects transgene expression when delivered by a lentivirus, or if lentiviral delivery is associated with any inflammation.

*Additional comments on duration of expression, cell types transduced and anti-vector immunity has been* ***added to lines 78-83****.* ***We have now shown by RT-qPCR and histology that there is no obvious inflammation induced by hACE2-lentivirus alone (Supplementary Figure 9, see lines 291-297)****. Please see response to reviewer #1 major comment #3.*

• Page 3, Line 63-65 and Lines 72-74. These statements could be updated with some discussion regarding a recent pre-print which suggests that some SARS-CoV-2 variants can infect mice using murine ACE2. This article also contains some investigation of binding residues for ACE2, so some interpretation and discussion of these data would be informative. I appreciate this pre-print is a new addition to the literature and was uploaded after this manuscript https://www.biorxiv.org/content/10.1101/2021.03.18.436013v1.full.pdf.

*This reference has been included on* ***line 75 and 182****.*

• Page 4, Line 80. Define GM.

*Genetically modified (GM) has been defined in the introduction* ***(line 69)****.*

• The statement on Page 7, Line 166-167 should be revised as the SARS-CoV-2 challenge doses in citations 10 and 43 differed 10-fold which may have contributed to differences in weight loss between the reported studies.

*This statement has been removed.*

• Page 11, Line 277-279. This statement should be elaborated on. Prior studies also showed that inhibition of Type I IFN did not affect SARS-CoV-2 replication but that it did affect the inflammatory response and resulted in pathological signs of severe viral pneumonia. The addition of histological data (as proposed in comments above) would add support to this for the lentivirus-hACE2 model.

*This statement is now elaborated on and* ***histology is now included (Figure 6F-H, lines 422-430)****. IFNAR^-/-^ mice had similar levels of bronchiolar sloughing, but had reduced leukocyte infiltration, consistent with the Ad5-hACE2 IFNAR^-/-^ model (Sun et al. 2020. Cell and Israelow et al. J Exp Med. 2020).*

• Page 14, Line 351-353. The authors should explain why long-term expression would be preferable and what sort of in vivo studies in mice would benefit from this.

*This is now included* ***(see lines 470-472)****.*

Reviewer #3: There are no minor issues of concern.
